# Supplementary material for: Minimizing the electrosorption of water from humid ionic liquids on electrodes
Source: Nat Commun. 2018 Dec 4;9:5222. doi: 10.1038/s41467-018-07674-0 (PMC6279789; doi:10.1038/s41467-018-07674-0)
Supplement: Supplementary file 1 — Supplementary Information [file 41467_2018_7674_MOESM1_ESM.pdf]

## Supplementary Information

# Minimizing the electrosorption of water from humid ionic liquids on electrodes

Bi et al.

## Table of Contents

Supplementary Note 1: Simulation setup ........ 3

Supplementary Note 2: Hydrophobicity/hydrophilicity and hygroscopicity of RTILs ........ 5

Supplementary Note 3: Interfacial structure of humid RTILs-electrodes interfaces ........ 8

Supplementary Note 4: Effects of the water concentration and electrode material ........ 11

Supplementary Note 5: Gold surface characterization ........ 14

Supplementary Note 6: Potential of mean force ........ 15

Supplementary Note 7: Orientation and H-bond of water adsorbed on the electrode ........ 17

Supplementary Note 8: Explorations on additional RTILs ........ 21

## Supplementary Note 1: Simulation setup

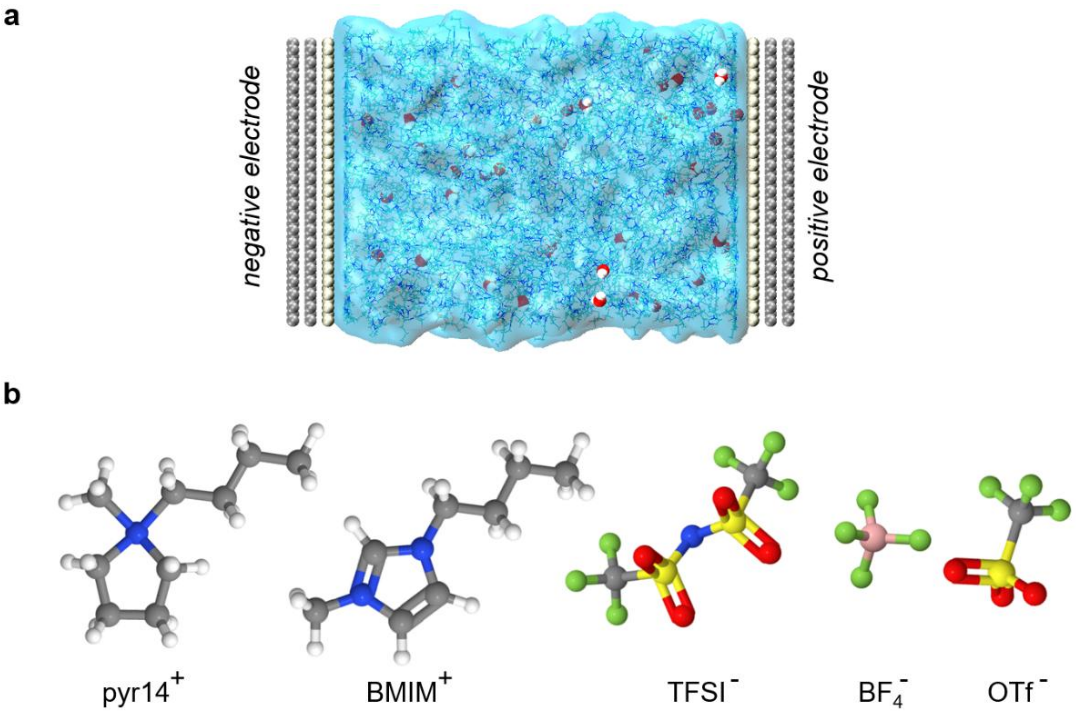


**Supplementary Figure 1 | Schematic of molecular dynamics (MD) simulation setup. a,** Snapshot of the humid ionic liquids between two planar electrodes. **b,** Molecular structure of cations and anions in room temperature ionic liquids (RTILs) [pyr14][TFSI], [BMIM][$BF_4$], [BMIM][TFSI] and [BMIM][OTf] used in this work.

**Supplementary Table 1 |** Number of electrode atoms, ion pairs and water molecules as well as water content in each molecular dynamics (MD) simulation system. The more-than-one values given in the right two columns indicate that such MD simulations were performed with different water content. Values in bold are for simulations presented in the main text.

| MD Simulation System | Electrode | Ion Pair | Water | ppm |
|---|---|---|---|---|
| Humid [pyr14][TFSI]-Au(111) | 418 | 456 | **30**/42 | **2796**/3910 |
| Humid [BMIM][$BF_4$]-Au(111) | 418 | 736 | 28/**46**/216 | 3021/**4953**/ 22838 |
| Humid [pyr14][TFSI]-graphite | 1144 | 456 | **30**/42 | **2796**/3910 |
| Humid [BMIM][$BF_4$]-graphite | 1144 | 736 | 28/**46** | 3021/**4953** |
| Humid [BMIM][TFSI]-Au(111) | 418 | 478 | 32 | 2865 |
| Humid [BMIM][OTf]-Au(111) | 418 | 624 | 50 | 4978 |

## Supplementary Note 2: Hydrophobicity/hydrophilicity and hygroscopicity of room temperature ionic liquids (RTILs)

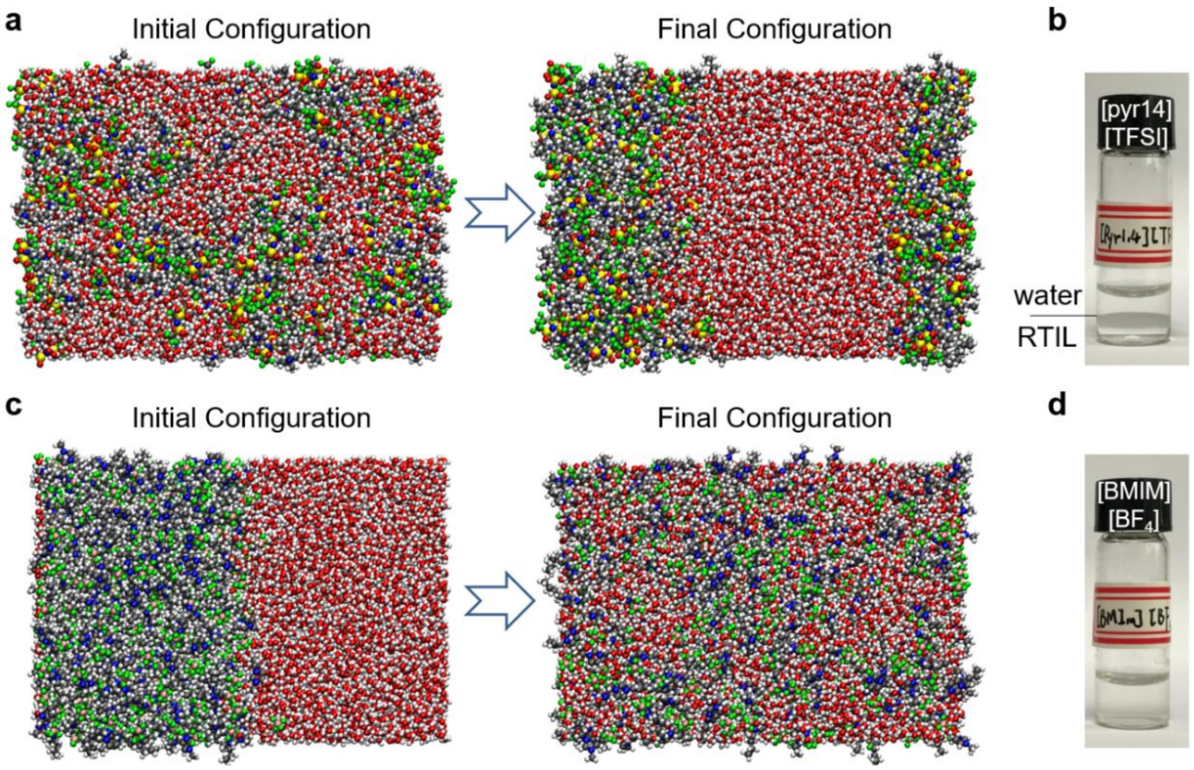


**Supplementary Figure 2 | Hydrophobicity and hydrophilicity of RTILs. a-b,** MD system snapshots (**a**) and experimental image (**b**) of water and hydrophobic RTIL [pyr14][TFSI]. **c-d,** MD system snapshots (**c**) and experimental image (**d**) of water and hydrophilic RTIL [BMIM][$BF_4$].

The terms of 'hydrophobic/hydrophilic' have been used in many published studies, in which they often refer to water miscibility with room temperature ionic liquids (RTILs).[1,2] For instance, Huddleston *et al.*[3] characterized a series of hydrophobic and hydrophilic RTILs, associated with water miscibility, by their equilibrated water content after being stored in contact with water. Herein, the miscibility of water with two major RTILs ([pyr14][TFSI] and [BMIM][$BF_4$]) was investigated by both MD simulations and experiments. As the system snapshots of MD simulation of water and RTILs shown in **Supplementary Figure 2**, MD simulations were all performed at 298K (Nosé-Hoover thermostat) and 1 atom (Berendsen barostat) in NPT ensemble, and the water-to-RTIL molar ratio is ~15:1. Specifically, MD simulation system of water/[pyr14][TFSI], starting from water "randomly mixed" with [pyr14][TFSI], mimics the process of demixing, in which water and RTIL are being separated

into two phases and eventually formed well-defined water-RTIL interfaces (**Supplementary Figure 2a** and **Supplementary Movie 1**). MD simulation system of water/[BMIM][$BF_4$], starting from water "initially separated" from [BMIM][$BF_4$], however, exhibits that water and RTIL could spontaneously mix with each other (**Supplementary Figure 2c** and **Supplementary Movie 2**). Therefore, MD simulations show that RTIL [pyr14][TFSI] is water-immiscible while RTIL [BMIM][$BF_4$] is miscible with water, which are in line with previous work[1,2].

The character of water miscibility with RTILs was further checked by experiments. Briefly, 2 mL water was added into 2 mL each of RTILs and the mixture was stirred. Then, the water/RTIL mixture was sealed and stored for two hours. The miscibility of water with [pyr14][TFSI] and [BMIM][$BF_4$] is determined optically. Although the two RTILs and water are colorless and transparent, the water/[pyr14][TFSI] interface can be observed in **Supplementary Figure 2b**, while the water/[BMIM][$BF_4$] mixture displays a homogeneous phase (**Supplementary Figure 2d**).

To examine the capability of RTILs [pyr14][TFSI] and [BMIM][$BF_4$] to attract and hold water molecules from surrounding environment, experiments were carried out under almost constant humidity and temperature (46.7±3.1% humidity, 299.8±0.5K for [pyr14][TFSI]; 49.4 ±2.3% humidity, 299.6±0.5K for [BMIM][$BF_4$]), and the change of the water content of RTILs with time was monitored to see their difference in 'hygroscopicity'. Specifically, after vacuum-dried at 353K for several hours in a glovebox filled with ultra-pure Ar (99.999%), RTILs were exposed to room environment to explore the water content change. Water concentration was measured by the Karl Fisher method[4,5] using an 831 KF Coulometer (Metrohm), meanwhile, both temperature and humidity were recorded. **Supplementary Figure 3** shows that RTIL [BMIM][$BF_4$] absorbs much more water from the air than [pyr14][TFSI]. After a 48-hour exposure to the air, the water content of [BMIM][$BF_4$] reaches to ~30000 ppm, which is approximately 8 times larger than that of [pyr14][TFSI] (~3500 ppm). Moreover, with one-week exposure the water content could stay at ~3900 ppm and ~33000 ppm for [pyr14][TFSI] and [BMIM][$BF_4$], respectively.

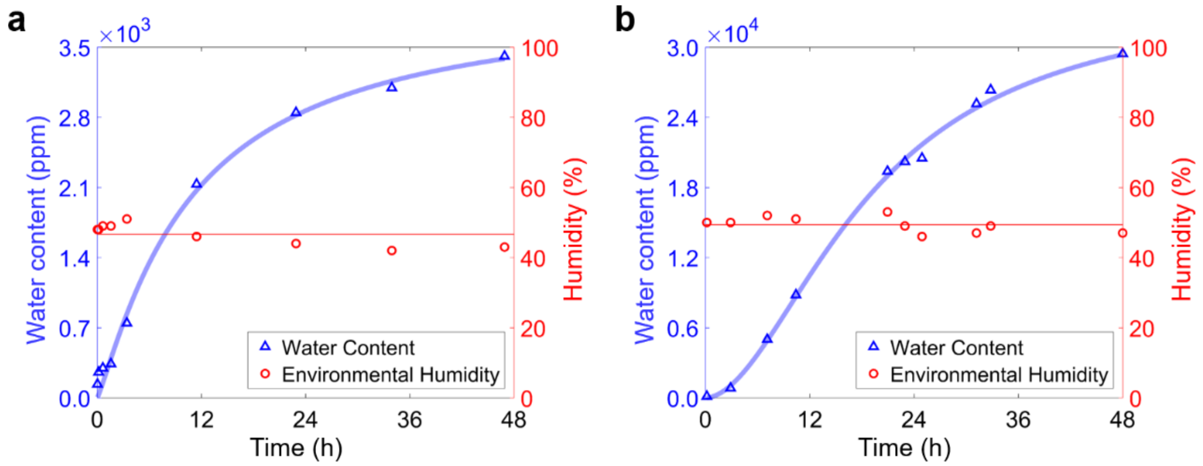


**Supplementary Figure 3 | Hygroscopicity of RTILs in humid air. a-b,** Water content in [pyr14][TFSI] (**a**) and [BMIM][$BF_4$] (**b**) as a function of time. Red solid lines represent the averaged humidity during the measurements; Blue lines are to guide the eyes.

## Supplementary Note 3: Interfacial structure of humid RTILs-electrodes interfaces

**Supplementary Figure 4** dipicts the ion distribution of humid [BMIM][$BF_4$] on gold and carbon electrodes. Similar to the results of [pyr14][TFSI] (**Figure 1** in the main text), both $BMIM^+$ and $BF_4^-$ ions show stronger adsorption on gold than on carbon electrodes, and $BMIM^+$ can still be adsorbed at the gold even under positive polarization.

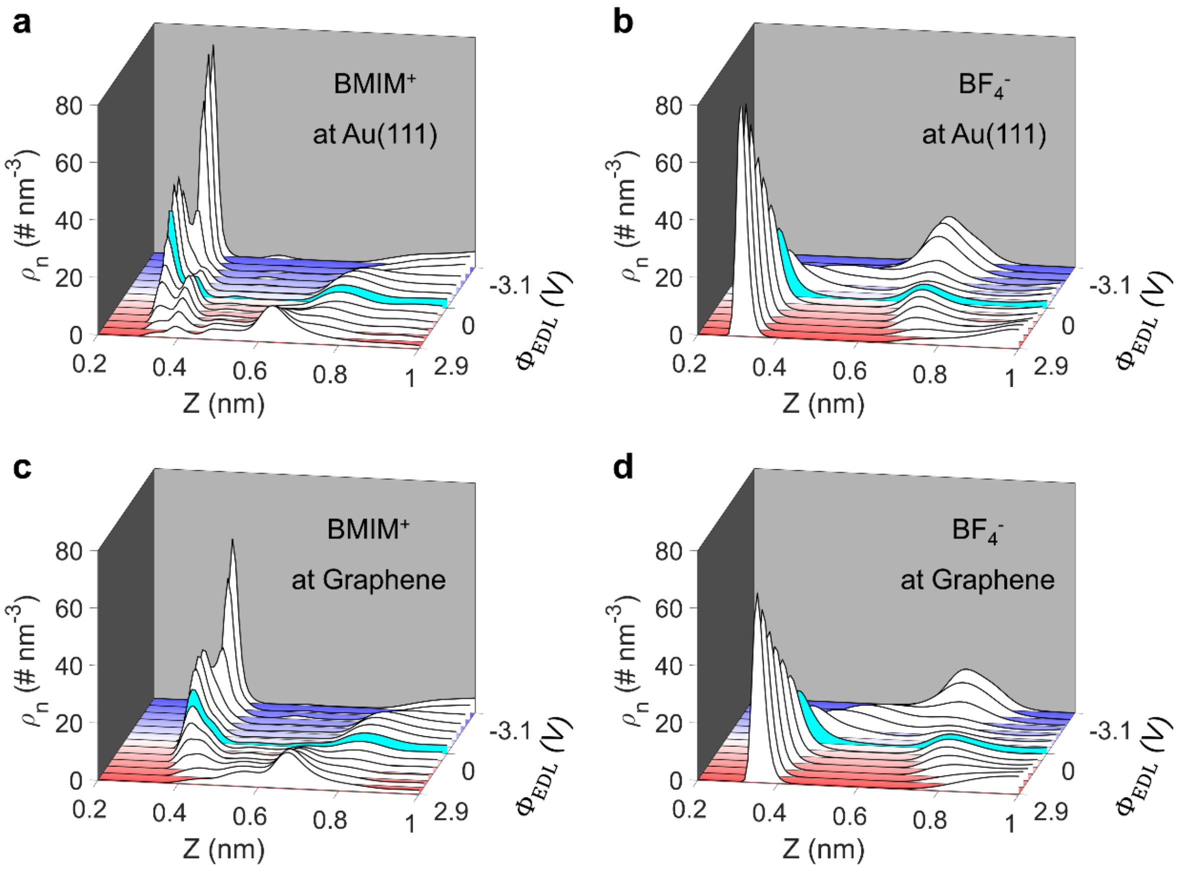


**Supplementary Figure 4 | Ion number density as a function of distance from the electrode with varying the EDL potential. a-d,** $BMIM^+$ (**a**) number density and $BF_4^-$ (**b**) number density on gold electrodes. $BMIM^+$ (**c**) number density and $BF_4^-$ (**d**) number density on carbon electrodes. Blue and red in floor color correspond to negative and positive potential respectively. Carbon and gold electrodes are located at Z = 0 nm, however, Z axis limits are specified from 0.2 nm to 1 nm for better illustration.

In **Supplementary Figure 5**, the ion distributions of humid [pyr14][TFSI] on gold and carbon electrodes are compared under the same surface charge density. One could see that gold is more ionophilic than carbon electrode in both netrual and electrified cases. From the **Supplementary Figure 6**, it can be observed that the water can be closer to the electrode surface than RTIL ions (based on their center of mass).

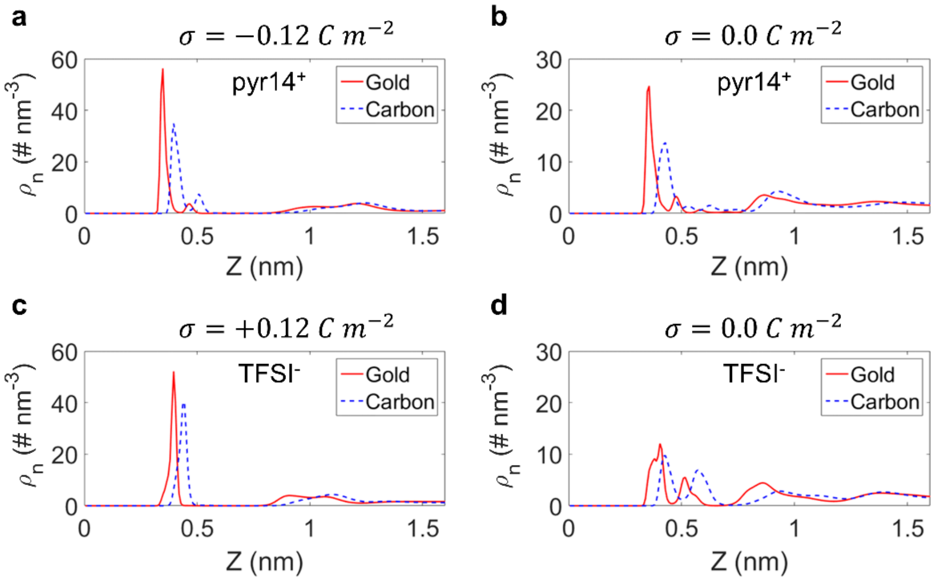


**Supplementary Figure 5 | Ion number density distribution for both gold and carbon electrodes under the same surface charge density σ. a-d,** $pyr14^+$ number density profiles under σ = -0.12 C $m^{-2}$ (**a**) and under σ = 0 C $m^{-2}$ (**b**). $TFSI^-$ number density profiles under σ = +0.12 C $m^{-2}$ (**c**) and under σ = 0 C $m^{-2}$ (**d**). Red solid lines and blue dashed lines represent results for gold and carbon electrodes, respectively.

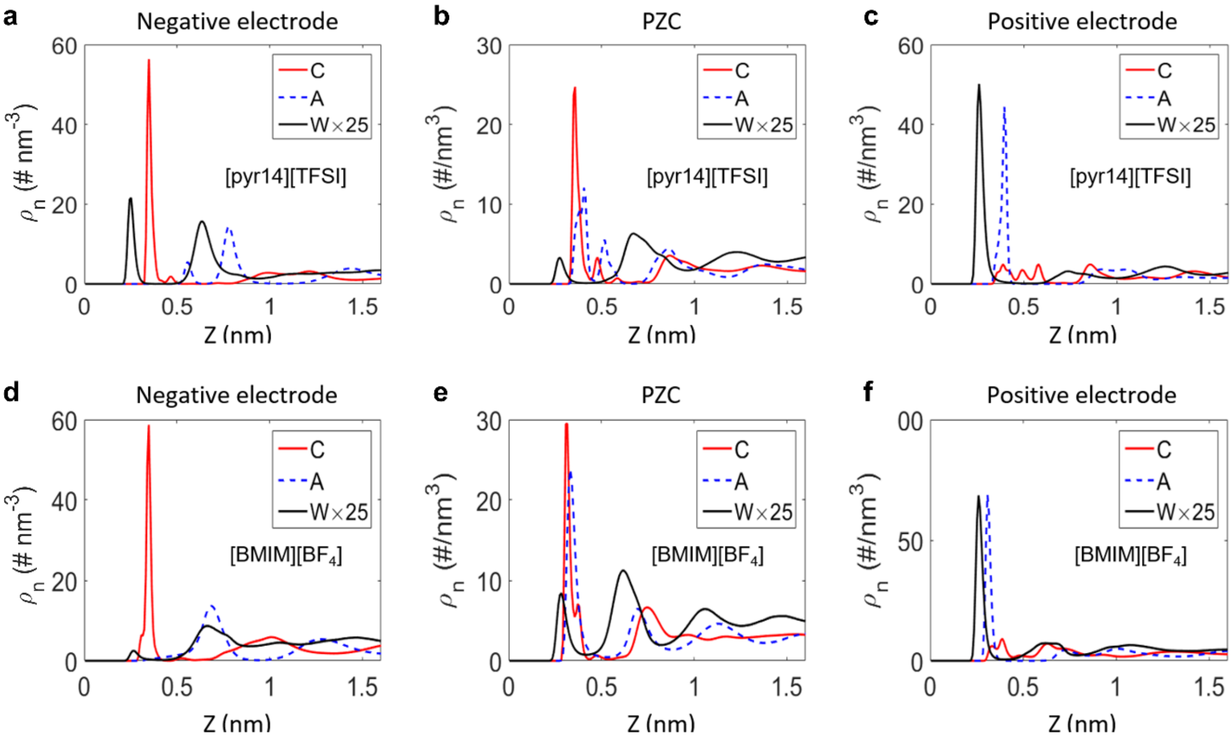


**Supplementary Figure 6 | Ion and water distributions in humid RTILs at gold electrodes. a-f,** Water and ion number density profiles of [pyr14][TFSI] (**a-c**) and [BMIM][$BF_4$] (**d-f**) on gold electrodes. Panels (**a**) and (**d**) correspond to the negative electrode when the applied potential between the two electrodes is 4V. Panels (**c**) and (**f**) are for positive electrode. Panels (**b**) and (**e**) are under potential of zero charge. In legends, C is cation; A is anion; W is water ( ×25 means that water density is multiplied by a factor of 25). The position of water or electrolyte ion is based on its center of mass.

## Supplementary Note 4: Effects of the water concentration and electrode material

The influence of water concentration on interfacial water adsorption was investigated for two major RTILs ([pyr14][TFSI] and [BMIM][$BF_4$]), *via* MD simulations with similar setup as described in **Methods** of the main text and **Supplementary Note 1**. For hydrophobic [pyr14][TFSI], a higher concentration of 3910 ppm is examined to compare with the water content used in the main text (2796 ppm), as 3910 ppm is close to the maximum water content that [pyr14][TFSI] could absorb from the humid air (**Supplementary Figure 3**). This concentration is also compatible with the water content used in experiments (3815 ppm, see **Figure 3a** in the main text). For hydrophilic [BMIM][$BF_4$], another two water concentrations (3021 and 22838 ppm) are considered on gold electrodes so that both lower and higher concentrations than that used in the main text (4953 ppm) can be examined. Then quite higher water content (22800 ppm) is tested in experiments on gold electrodes as well (see **Figure 3b** in the main text).

Revealed by MD simulation, the effects of water concentration on the trend of electrosorption of water *vs* EDL potential, $\phi_{EDL}$, are not significant for these two studied RTILs on both gold and carbon electrodes (**Supplementary Figures 7a-b**). For hydrophobic [pyr14][TFSI], increasing water concentration from 2796 to 3910 ppm does not change the water electrosorption curves much, regardless of electrode materials. At both studied concentrations, an enrichment of water electrosorption could be seen at both cathode and anode surfaces under high polarization. For the hydrophilic [BMIM][$BF_4$], either increasing or decreasing the water concentration does not alter the trend of interfacial water electrosorption with respect to EDL potential. Even if the water content reaches a very high level (22838 ppm), depletion of water could still be observed at the negatively charged electrode. These results indicate that under the certain limit of the concentration that dry RTILs could reach at normal environment, water concentration plays a minor role in the water electrosorption on the electrified electrode surface.

The weak dependence of water concentration on interfacial water adsorption could be tested by MD simulations for humid [pyr14][TFSI] and [BMIM][$BF_4$] at electrified carbon electrodes (**Supplementary Figures 7c-d**). Furthermore, compared with gold electrodes (**Supplementary Figures 7a-b**), the electrode type is found to play a minor role on the interfacial water adsorption.

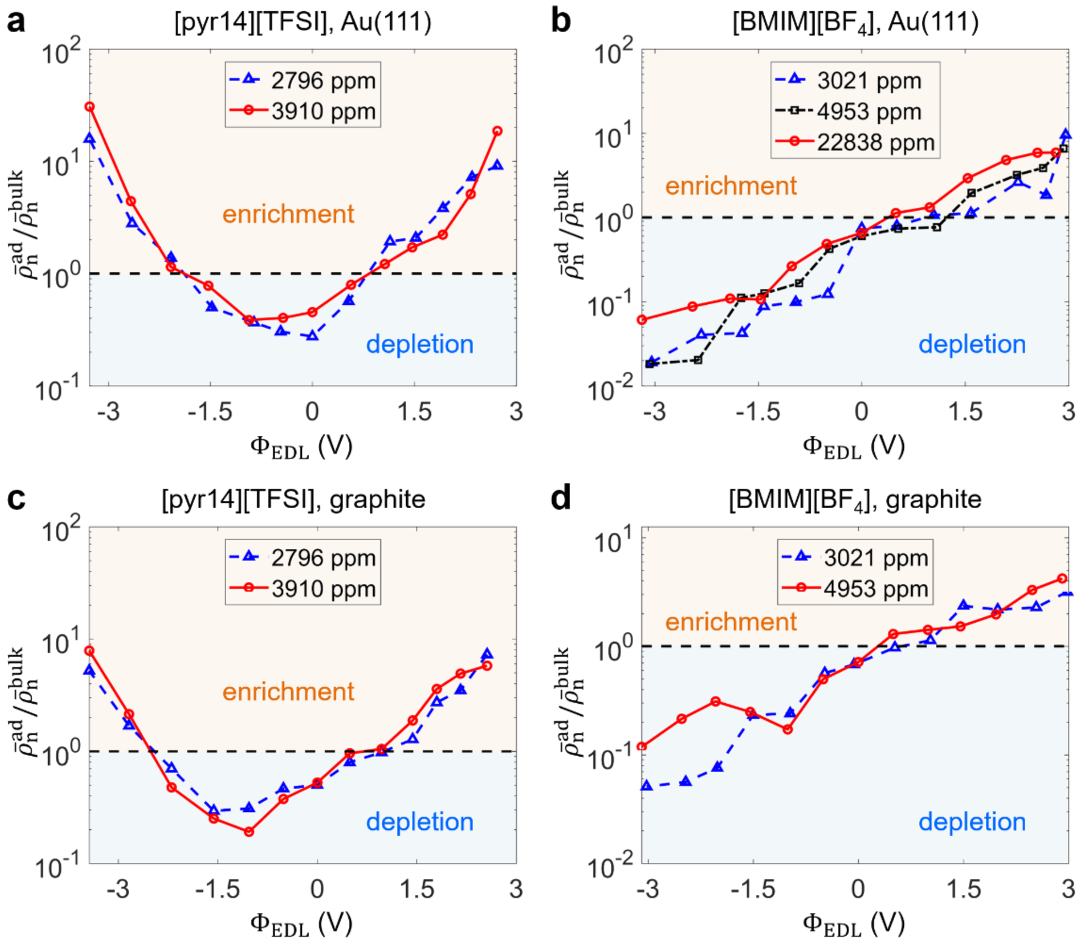


**Supplementary Figure 7 | Electrosorption of water from RTILs with different water contents on gold and carbon electrodes.** Panels (**a**) and (**b**) show water adsorption from humid [pyr14][TFSI] and [BMIM][$BF_4$] with different water content on gold electrodes, respectively; Panels (**c**) and (**d**) are for these two humid RTILs on carbon electrodes.

To verify this weak impact from electrode type revealed by MD simulation, the effect of water sorption on electrochemical activity at carbon electrode was investigated by using **H**ighly **O**riented **P**yrolytic **G**raphite (HOPG) as working electrode. The surface structure of HOPG is very close to the one used in the simulation model. A Pt wire and an Ag wire serve as counter electrode and reference electrode, respectively.

Cyclic voltammograms of HOPG in humid RTIL [pyr14][TFSI] were shown in **Supplementary Figure 8a**. It can be seen that when the water content increased from 570 to 3815 ppm, reduction current abruptly increased at around -0.7 V, which was not observed in [pyr14][TFSI] containing 570 ppm water, resulting in a narrowed electrochemical window. Since all experimental conditions except water content was kept unchanged, the decrease of electrochemical window with the increase of water content should be ascribed to the reduction of water. However, for hydrophilic [BMIM][$BF_4$], similar CV curves were obtained when water content increased from 590 to 3915 ppm (see **Supplementary Figure 8b**). No significant reduction current was observed and the electrochemical window is almost the same under different water content.

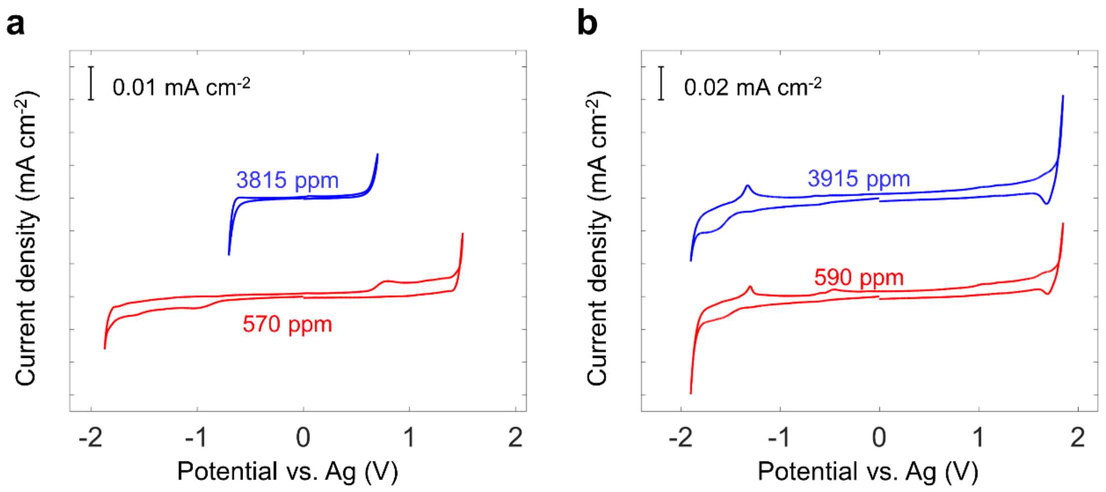


**Supplementary Figure 8 | The effect of water sorption on electrochemical activity on carbon electrode. a-b,** Cyclic voltammograms of HOPG in hydrophobic [pyr14][TFSI] (**a**) and hydrophilic [BMIM][$BF_4$] (**b**) under different water contents. Scan rate: 100 mV/s.

Therefore, both modeling and experiment demonstrate that carbon electrode presents the very similar feature as gold electrode, concerning the effect of water sorption on electrochemical activity in humid RTILs.

## Supplementary Note 5: Gold surface characterization

Au(111) surface was imaged by Atomic Force Microscope (AFM, Agilent 5500). It can be seen that the Au(111) surface is flat and a step can be observed, which crosses Au(111) terrace. Sectional analysis was performed to measure the height of the Au step, which gives the height to be about 0.25 nm, indicating that the surface of Au(111) electrode we used is atomically flat with the existence of single atomic step on the terrace.

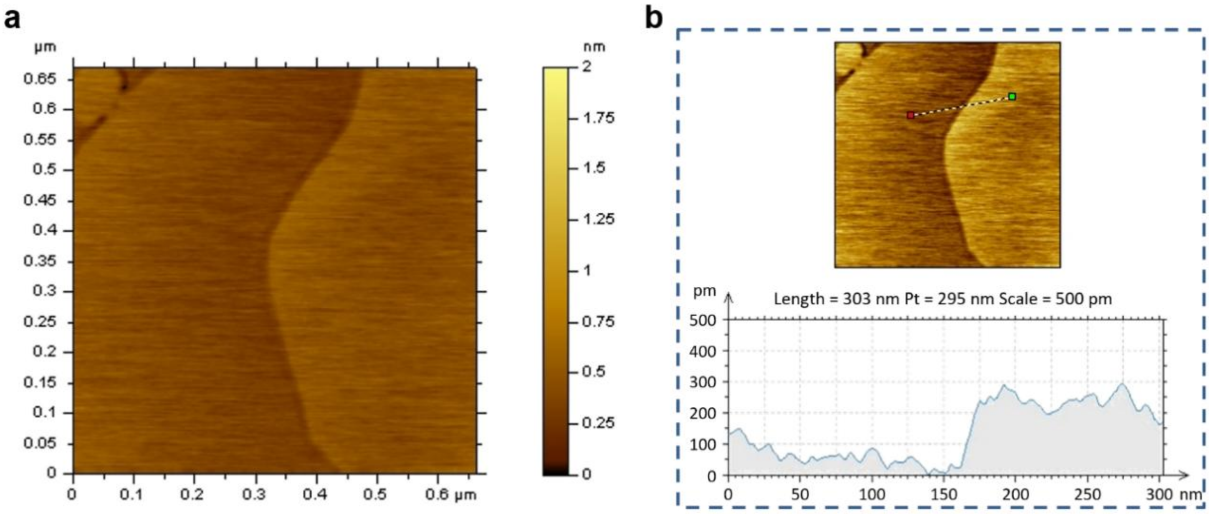


**Supplementary Figure 9 | AFM images of gold electrode. a,** AFM image of Au(111) electrode. **b**, Sectional analysis for characterizing the height of Au step in the AFM image.

## Supplementary Note 6: Potential of mean force

Herein, we show PMF results of water in RTILs [pyr14][TFSI] and [BMIM][$BF_4$] under applied potential of 2 V as well as PMFs of carbon electrodes at the applied potential of 4 V (see **Supplementary Figures 10** and **11**, respectively). Based on PMF curves on gold electrodes under a different applied potential (2 V), it can be seen that the free energy of water in [BMIM][$BF_4$] already lies higher at the negatively charged electrode than that of water in the bulk region (**Supplementary Figure 10c**). In addition, compared with **Figure 4** in the main text for PMFs under 4 V, it could be observed that increasing applied potential (from 2 to 4 V) could lead to a more negative free energy of water in [pyr14][TFSI] near both negative and positive electrodes as well as in [BMIM][$BF_4$] near positive electrode. However, it results in a higher free energy of interfacial water in [BMIM][$BF_4$] under the negative polarization. This can help to explain why in humid [BMIM][$BF_4$] depletion of water becomes more distinct as the EDL potential goes more negative.

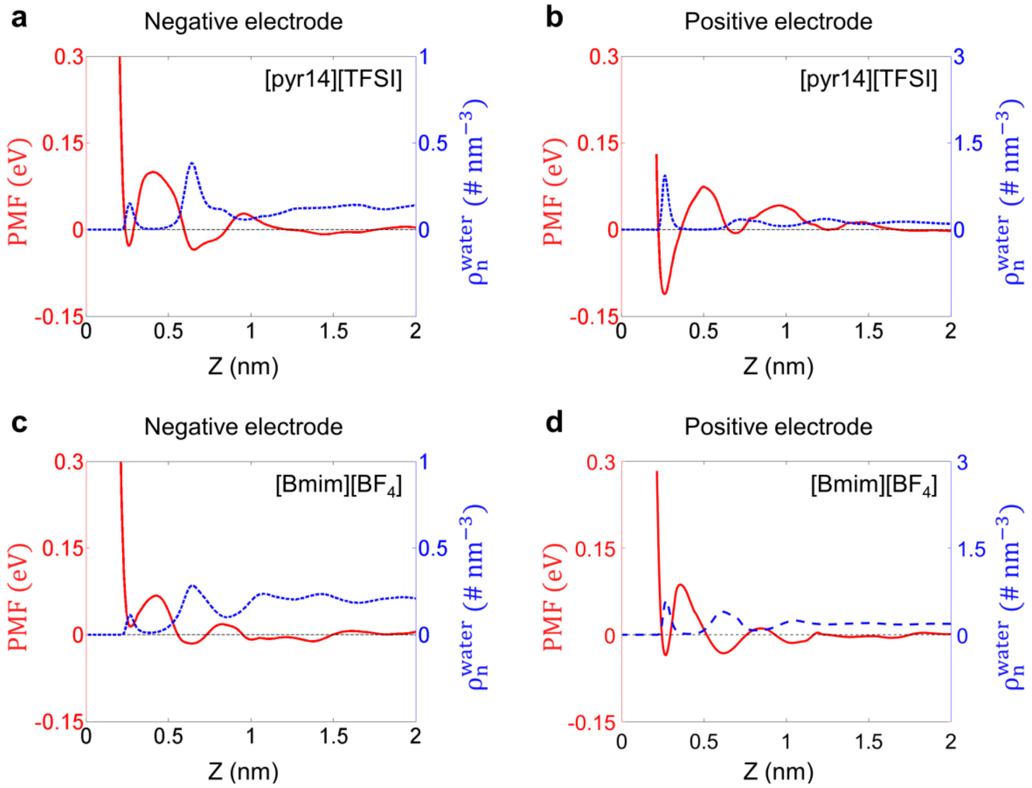


**Supplementary Figure 10 | The propensity for water electrosorption from hydrophobic and hydrophilic RTILs at gold electrodes under an applied potential of 2 V. a-d,** Correlation between the distributions of water density (dashed blue lines, right blue y-axis) and PMF (solid red lines, left red y-axis), for [pyr14][TFSI] near negative (**a**) and positive(**b**) electrodes, and [BMIM][$BF_4$] near negative (**c**) and positive (**d**) electrodes.

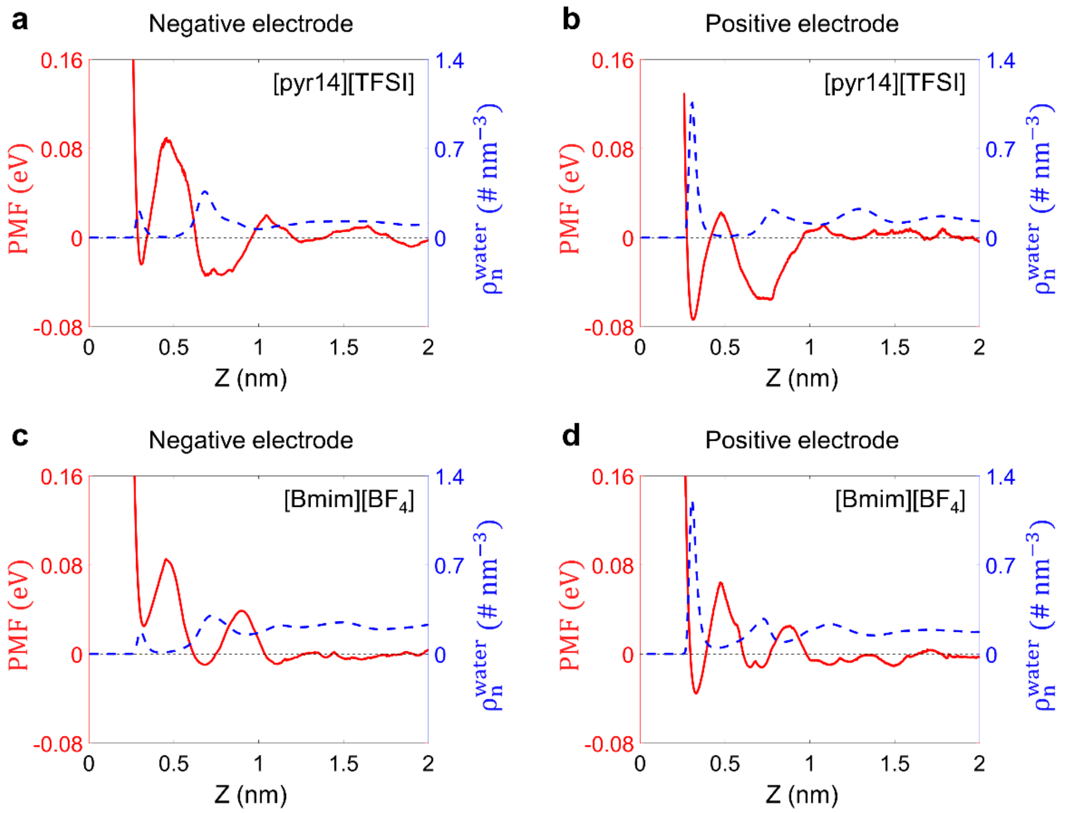


**Supplementary Figure 11 | The propensity for water electrosorption from hydrophobic and hydrophilic RTILs at carbon electrodes under a applied potential of 4 V. a-d,** Correlation between the distributions of water density (dashed blue lines, right blue y-axis) and PMF (solid red lines, left red y-axis), for [pyr14][TFSI] near negative carbon electrode (**a**) and positive electrode (**b**), and [BMIM][$BF_4$] near negative electrode (**c**) and positive electrode (**d**).

Similar to the gold electrode (**Figure 4** in the main text**)**, PMF curves on carbon electrodes correspond well to the distribution of water in double layers. For hydrophobic [pyr14][TFSI], a clear minimum of PMF exists very close to both negatively and positively charged electrodes (about -0.024 eV at ~ 0.31 nm near negative electrode, and about -0.074 eV at ~ 0.31 nm near positive surface). This reveals that water in hydrophobic [pyr14][TFSI] tends to accumulate near both electrodes, resulting the enrichment of water at the interfacial region. For hydrophilic [BMIM][$BF_4$], however, one can see a distinct positive potential well near the negative electrode (about +0.025 eV at ~0.32 nm), which indicates that water is redundant to stay there. These findings further confirm that the electrode type has a secondary impact on water electrosorption, as it does not change the shapes of PMF curves and water distributions much.

## Supplementary Note 7: Orientation and H-bond of water adsorbed on the electrode

To scrutinize the structure of interfacial water, both dipole and normal orientations of interfacial water molecules, incorporated in hydrophobic [pyr14][TFSI] and hydrophilic [BMIM][$BF_4$], are delved when the applied potential between two electrodes is 4V. Through this work, water dipole orientation, $\theta_{dipole}$, is defined as the angle formed by the water vector and the normal of electrode surface; water normal orientation, $\theta_{normal}$, is defined as the angle formed between the normal of water plane and the normal of electrode surface. As shown in **Supplementary Figure 12**, one can observe that interfacial water molecules in [pyr14][TFSI] and [BMIM][$BF_4$] exhibit different orientation near negatively charged electrodes, while they are similar at positive side. These results can help to understand the propensity of water electrosorption from hydrophobic and hydrophilic RTILs.

Specifically, at negatively charged gold electrodes, the dipole orientation (the highest probability at 152 °) of interfacial water molecules in hydrophobic [pyr14][TFSI] as well as their normal orientation (peak located at 90 °) indicates that most water tends to be vertical to the electrode surface with its two hydrogen atoms both approaching to the surface. Although water in hydrophilic [BMIM][$BF_4$] is also vertical to the electrode, however, it is more sideling than in [pyr14][TFSI] as one of its hydrogen atom is closer to the surface than the other (the dipole orientation shows two probability peak located at 90 ° and 154 °, respectively). Near positively charged electrodes, for both RTILs and electrodes, the normal orientations (which means the distribution of angle formed between the normal of water plane and the normal of electrode surface), showing two peaks located at 16 ° and 164 ° as well as the dipole orientations with peak location at around 75 °, depict that most water adopts a configuration parallel to the electrode. It also implies that both electrode type and the hydrophobic/hydrophilic nature of RTILs have weak influence on the water orientation at positively charged electrodes.

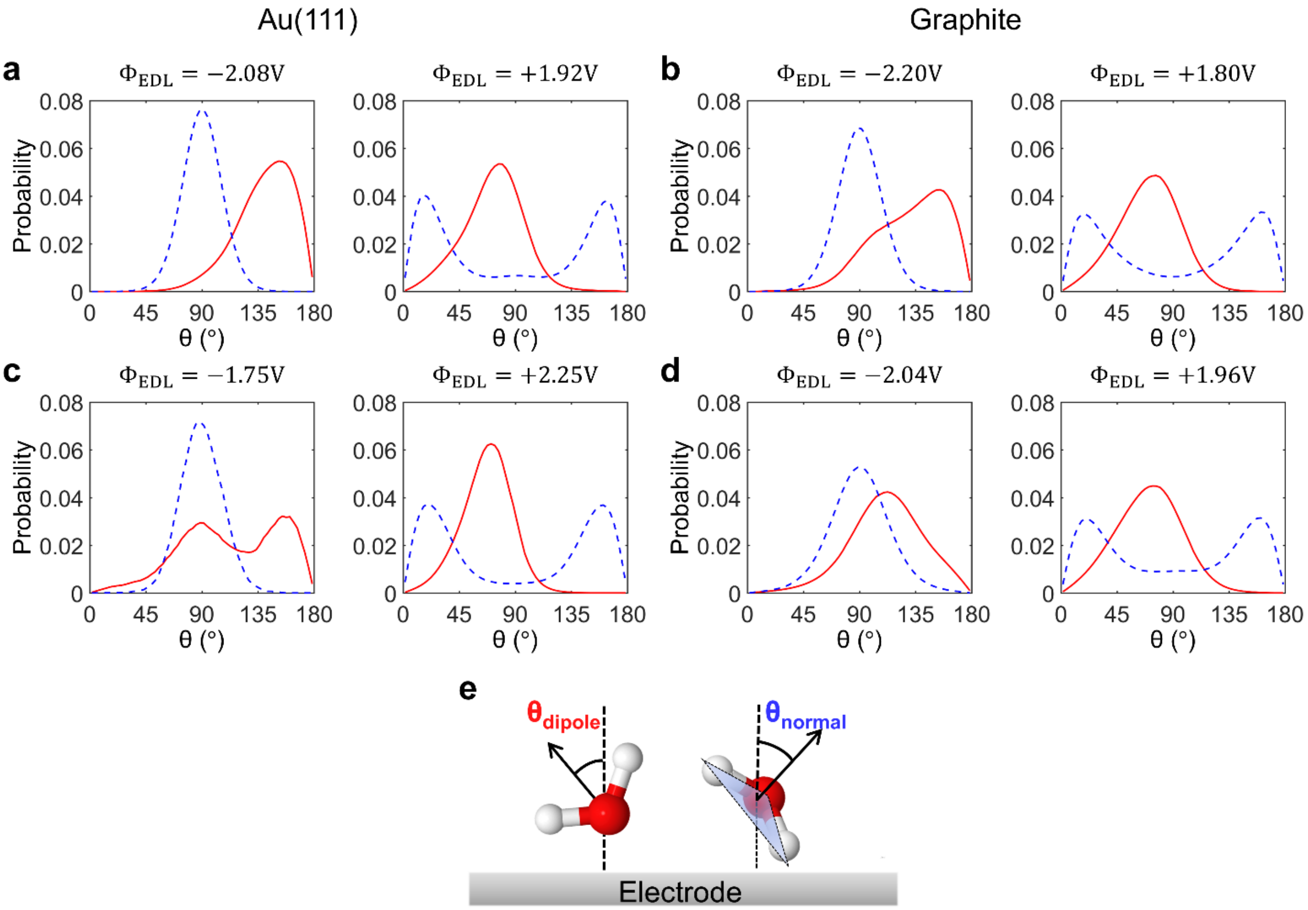


**Supplementary Figure 12 | Orientation of interfacial water. a-b,** Water orientation with hydrophobic [pyr14][TFSI] at gold (**a**) and carbon (**b**) electrodes. **c-d,** Water orientation with hydrophilic [BMIM][$BF_4$] at gold (**c**) and carbon (**d**) electrodes. **e,** The cartoon of orientational angles. Red solid and blue dashed lines represent water dipole and normal orientations, respectively. The EDL potential is labeled on the top of each figure, and the applied potential between two electrodes is 4V.

As a result, changing electrode type from gold to carbon does not alter much about the trend of these observations, which further suggests the minor role of electrode type in water electrosorption.

How would the hydrophobic/hydrophilic nature, especially the cation/anion type, affect the structure of interfacial water? To answer this question, we analyze the number of hydrogen bonds (H-bonds) that one interfacial water molecule could *averagely* form with surrounding cations/anions with respect to EDL potential. In this work, for a cation we define an H-bond between a hydrogen atom of cation and an oxygen atom of water to be formed if the distance between them, $R_c$, is shorter than 0.35 nm and the angle of hydrogen–carbon–oxygen, $\theta_c$, (each carbon atom in cation could be donor and oxygen atom

in water is accepter) is less than 30 degrees[6]. For an anion, an H-bond is determined between a hydrogen of water and an electronegative atom of anion (acting as acceptor, here are fluorine, nitrogen or oxygen atom).

As exhibited in **Supplementary Figure 13**, under zero potential, the number of H-bonds formed between water and anion is much larger than that of water and cation, regardless of electrode type and RTIL type.

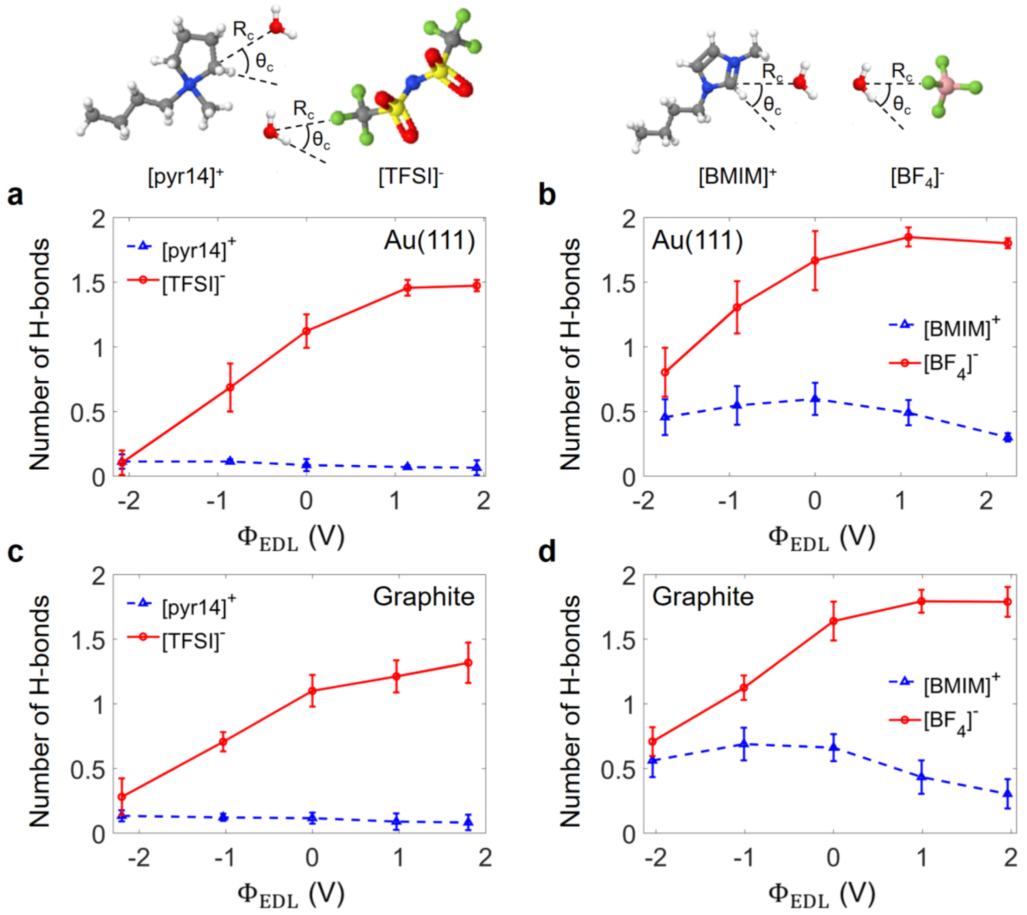


**Supplementary Figure 13 | Hydrogen bonds formed by RTIL ions and interfacial water as a function of applied potential. a-b,** Number of H-bond by cations/anion in [pyr14][TFSI] (**a**) and in [BMIM][$BF_4$] (**b**) with interfacial water molecules at gold electrodes. **c-d,** Number of H-bond by cations/anions in [pyr14][TFSI] (**c**) and in [BMIM][$BF_4$] (**d**) with interfacial water molecules at carbon electrodes. The images above panel (**a**) and (**b**) show the schematics of H-bond by water molecules with cations and anions in [pyr14][TFSI] and [BMIM][$BF_4$], respectively.

When the electrode surface becomes more negatively electrified, for hydrophobic [pyr14][TFSI] the number of water–$pyr14^+$ H-bonds remains a very small value (**Supplementary Figure 13a**), and that of water–$TFSI^-$ H-bonds decreases dramatically (e.g., from 1.12 to 0.10 on gold electrode, **Supplementary Figure 13c**). The scenario of

[BMIM][$BF_4$] is much different (**Supplementary Figures 13b and 13d**): water and $BF_4^-$ still form more H-bonds than water with $BMIM^+$ even in $BMIM^+$-rich double layers (for instance, 0.8 vs. 0.46 on gold electrode). These findings could help to understand why hydrophobic [pyr14][TFSI] and hydrophilic [BMIM][$BF_4$] could lead to very different water orientation near negative polarization (**Supplementary Figure 12**), that is, 1) in the interfacial region under negative polarization, hydrogen bonding of water–[BMIM][$BF_4$] is much stronger than water–[pyr14][TFSI]; 2) Although $BF_4^-$ ions are squeezed away from the negatively charged electrode, they are still strongly associated with interfacial water molecules in terms of formed H-bonds so that hydrogen atoms of water are attracted to point to the fluorine atoms.

When the EDL potential gets more positive, the number of water–anion H-bonds increases a little bit, while each water could form fewer hydrogen bonds with cations (for [BMIM][$BF_4$]) or maintain at a quite low level (for [pyr14][TFSI]). This can be ascribed to the anion accumulation and cation removal in EDLs near positively charged electrodes (see **Figure 1** in the main text and **Supplementary Figure 4**). Illustrated by the normal orientation of the interfacial water under positive polarization shown in the **Supplementary Figure 12**, water tends to lie on the electrode surface, as water forms strong hydrogen bonding with the surrounding anions.

Moreover, either cation or anion in hydrophilic [BMIM][$BF_4$] could have stronger hydrogen bonding with water than in hydrophobic [pyr14][TFSI], over the whole potential range. This observation is in line with previous studies reporting that water molecules would be associated with cations, however, they have stronger interaction with anions, which also account for why anions dominate the miscibility of RTILs.[7,8]

## Supplementary Note 8: Explorations on additional RTILs

To examine the effects of RTIL type as well as the ion type on the observations revealed by both MD simulations and electrochemical measurements of hydrophobic [pyr14][TFSI] and hydrophilic [BMIM][$BF_4$], another two RTILs ([BMIM][TFSI] and [BMIM][OTf]) were selected, since literatures[9–11] already reported that [BMIM][TFSI] is hydrophobic (water inmiscible) while [BMIM][OTf] is hydrophilic (water miscible). For this examination, we fucosed on gold electrodes.

Firstly, in MD simulations we investigated water electrosorption versus EDL potential at humid RTILs-gold interfaces (**Supplementary Figure 14**). It can be found that: 1) for hydrophobic [BMIM][TFSI], enrichment of interfacial water electrosorption occurs as the electrode becomes more polarized; 2) for hydrophilic [BMIM][$BF_4$], the depletion of water at the interficial region gets more obviously as the EDL potential goes more negative.

These findings, with the same trend of observations for hydrophobic [pyr14][TFSI] and hydrophilic [BMIM][$BF_4$] (**Figure 2** in the main text and **Supplementary Figure 7**), further affirm the conclusion that hydrophilic RTILs, rather than hydrophobic ones, could actually prevent water electrosorption on negatively charged electrode.

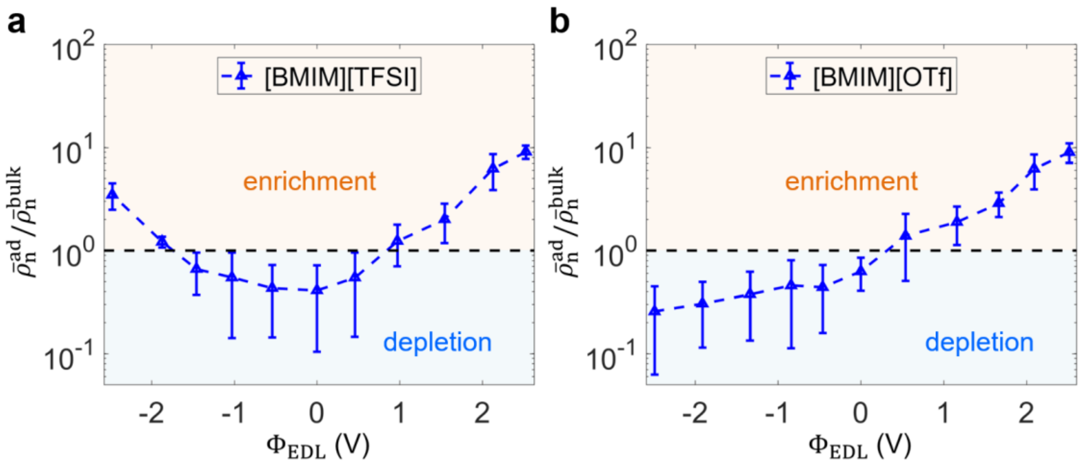


**Supplementary Figure 14 | Electrosorption of water from humid RTILs on gold electrodes**. Panels (a) and (b) show water adsorption on Au(111) from humid [BMIM][TFSI] and [BMIM][OTf], respectively. The relative enrichment or depletion of water molecules in the first interfacial layer is depicted by the ratio of averaged number density of water in this layer to that of water in the bulk region. Enrichment (depletion) zone corresponds to a higher (lower) water density than in the bulk.

Secondly, CV measurements were carried out for hyrophobic [BMIM][TFSI] and hydrophilic [BMIM][OTf]) on Au(111) electrodes as well. For hyrophobic [BMIM][TFSI], when water content increased from 410 to 3099 ppm, the lower limit of electrochemical window moved from around -2.2 to -1.7 V (**Supplementary Figure 15a**). The electrochemical behavior that the increase of water content decreases the electrochemical window is consistent with that of hyrophobic [pyr14][TFSI] (**Figure 3a** in the main text). As expected, for hydrophilic [BMIM][OTf], similar CV curves were obtained when water content is increased from 1230 to 3966 ppm (**Supplementary Figure 15b**). No obvious change occurs in the electrochemical window, i.e., the effect of water sorption on electrochemical activity in hydrophilic [BMIM][OTf] is consistent with that in hydrophilic [BMIM][$BF_4$] on Au(111) electrode (**Figure 3b** in the main text). These experimental results further support the conclusion drawn from MD simulations.

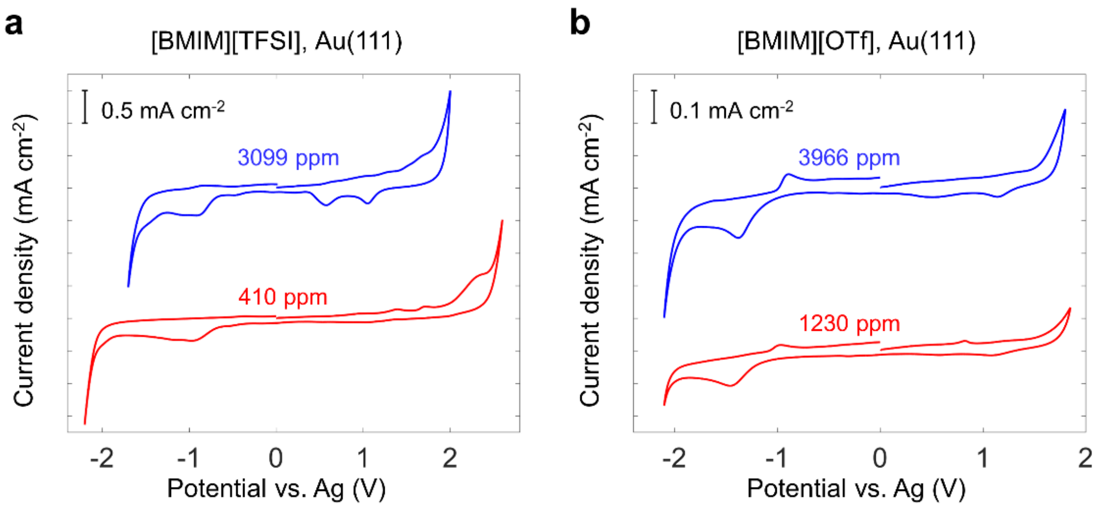


**Supplementary Figure 15 | The effect of water sorption on electrochemical activity on gold electrodes. a-b,** Cyclic voltammograms of Au(111) in hydrophobic [BMIM][TFSI] (**a**) and hydrophilic [BMIM][OTf] (**b**). Scan rate: 100 mV/s.

Note that the anions mostly determine water miscibility with RTILs, however, cations can still impact the hydrophobicity and hydrogen bonding.[3,12,13] Herein by an overall comparison of RTILs with the same type of anion ([pyr14][TFSI] *vs* [BMIM][TFSI]) or the same cation ([BMIM][TFSI] *vs* [BMIM][$BF_4$]), we probe into the effects of ion type (both cations and anions) on the interfacial water.

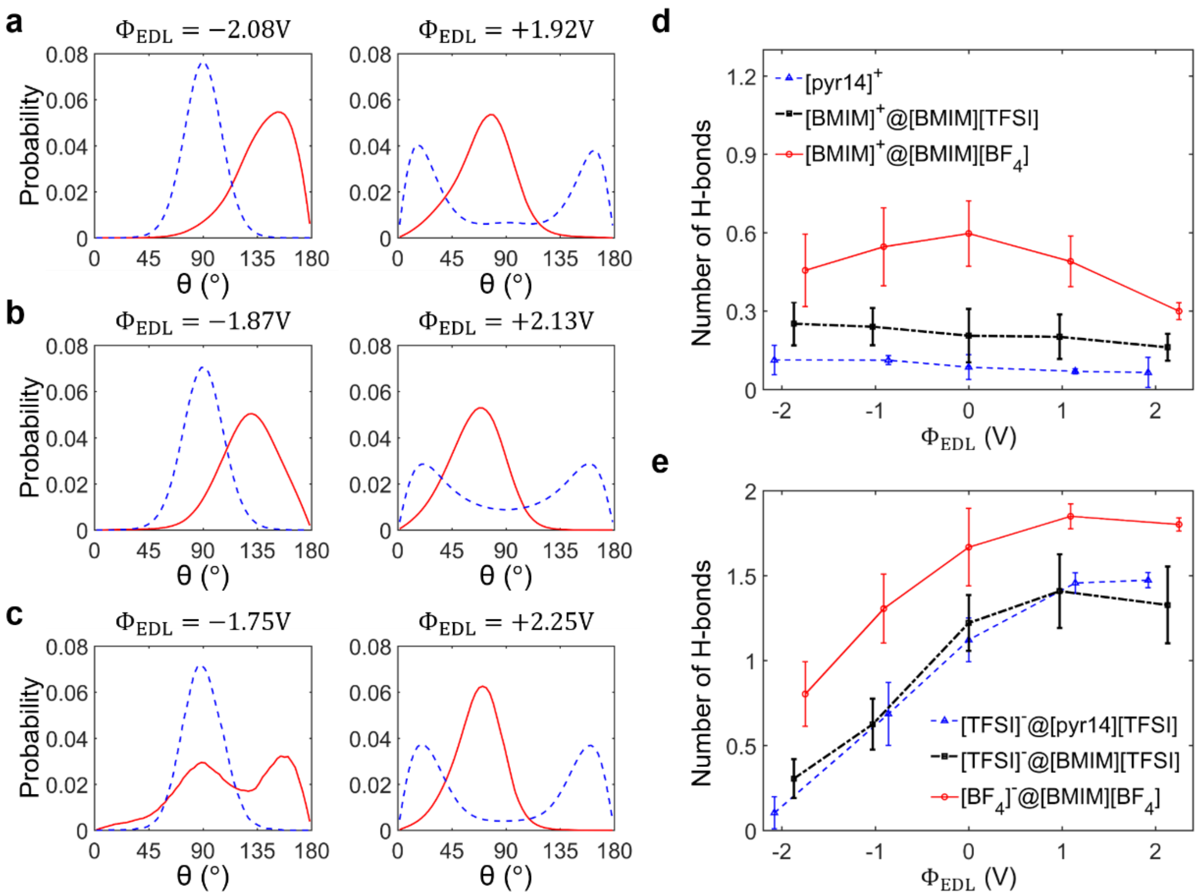


**Supplementary Figure 16 | Influence of ion type on interfacial water at gold electrodes. a-c,** Orientation of the interfacial water for RTILs [pyr14][TFSI] (**a**), [BMIM][TFSI] (**b**), and [BMIM][$BF_4$] (**c**) under an applied potential of 4 V. **d-e,** H-bonds formed by RTIL cations (**d**) and anions (**e**) with interfacial water as a function of applied potential. The orientation angle and the H-bond have the same definition as **Supplementary Figures 12** and **13**, respectively. The data for RTILs [pyr14][TFSI] and [BMIM][$BF_4$] are same as **Supplementary Figures 12** and **13**, just for comparison with [BMIM][TFSI] in convenience.

**Supplementary Figures 16a-c** display the comparison between the orientations of water molecules located at interfacial region from humid RTILs [pyr14][TFSI], [BMIM][TFSI] and [BMIM][$BF_4$] on gold electrodes, respectively. Under negative polarization, hydrophobic [BMIM][TFSI] has a similar orientation scheme of interfacial water to that for hydrophobic [pyr14][TFSI], with a peak shift of the probability curve of the dipole orientation (from 152 ° to 130 °). This suggests that cation does affect the structure of interfacial water. However, it can be seen that the interfacial water orientation in hydrophilic [BMIM][$BF_4$] differs a lot from that in two hydrophobic RTILs, though it has the same cation as hydrophobic [BMIM][TFSI], suggesting a dominant role of anion type on water adsorption.

**Supplementary Figures 16d-e** compare the number of H-bonds of water with these three RTILs under different EDL potential. When changing cation $pyr14^+$ to $BMIM^+$, water does interact a little bit stronger with $BMIM^+$ in [BMIM][TFSI] than with $pyr14^+$ in [pyr14][TFSI]. However, the number of water–$TFSI^-$ H-bonds is similar and small for in these two hydrophobic RTILs. By contrast, with replacing $TFSI^-$ in [BMIM][TFSI] to $BF_4^-$, not only water could form more H-bonds with $BF_4^-$ than $TFSI^-$ over the whole potential range, but also the hydrogen bonding interaction is enhanced between water and $BMIM^+$.

According to previous studies,[3,9] even if the water miscibility of RTIL is determined by anion type, cation can influence the hydrophobicity (i.e., immiscibility, but the maximum water content may differ). For example, for imidazolium-based RTILs, increasing the alkyl chain length from buty1 to hexyl to octyl could increase their hydrophobicity;[3] with the same anion, RTILs with pyridinium cation is more hydrophobic than RTILs with imidazolium cation.[9] Therefore, beyond consistence with these literature, our simulation results enucleate that: 1) the type of cation does affect the hydrogen bonding between water and cation; 2) the effect of anions on the number of H-bonds is much more significant than of cations; 3) altering anion from hydrophobic one to a hydrophilic type would enhance the water–cation hydrogen bonding as well.

## Supplementary References


1. Rivera-Rubero, S. & Baldelli, S. Influence of water on the surface of hydrophilic and hydrophobic room-temperature ionic liquids. *J. Am. Chem. Soc.* **126,** 11788–11789 (2004).
2. Gutowski, K. E. *et al.* Controlling the aqueous miscibility of ionic liquids: aqueous biphasic systems of water-miscible ionic liquids and water-structuring salts for recycle, metathesis, and separations. *J. Am. Chem. Soc.* **125,** 6632–6633 (2003).
3. Huddleston, J. G. *et al.* Characterization and comparison of hydrophilic and hydrophobic room temperature ionic liquids incorporating the imidazolium cation. *Green Chem.* **3,** 156–164 (2001).
4. Westrich, H. R. Determination of water in volcanic glasses by Karl-Fischer titration. *Chem. Geol.* **63,** 335–340 (1987).
5. Smith, A. M. *et al.* Monolayer to bilayer structural transition in confined pyrrolidinium-based ionic liquids. *J. Phys. Chem. Lett.* **4,** 378–382 (2013).
6. Vlcek, L. *et al.* Electric Double layer at metal oxide surfaces: Static properties of the cassiterite−water interface. *Langmuir* **23,** 4925–4937 (2007).
7. D’Angelo, P., Zitolo, A., Aquilanti, G. & Migliorati, V. Using a combined theoretical and experimental approach to understand the structure and dynamics of imidazolium-based ionic liquids/water mixtures. 2. Exafs spectroscopy. *J. Phys. Chem. B* **117,** 12516–12524 (2013).
8. Porter, A. R., Liem, S. Y. & Popelier, P. L. A. Room temperature ionic liquids containing low water concentrations—a molecular dynamics study. *Phys. Chem. Chem. Phys.* **10,** 4240–4248 (2008).
9. Cao, Y., Chen, Y., Sun, X., Zhang, Z. & Mu, T. Water sorption in ionic liquids: Kinetics, mechanisms and hydrophilicity. *Phys. Chem. Chem. Phys.* **14,** 12252–12262 (2012).
10. Baj, S., Chrobok, A. & Słupska, R. The Baeyer–Villiger oxidation of ketones with bis(trimethylsilyl) peroxide in the presence of ionic liquids as the solvent and catalyst. *Green Chem.* **11,** 279–282 (2009).
11. Malik, R. S., Tripathi, S. N., Gupta, D. & Choudhary, V. Novel anhydrous composite membranes based on sulfonated poly (ether ketone) and aprotic ionic liquids for high temperature polymer electrolyte membranes for fuel cell applications. *Int. J. Hydrogen Energy* **39,** 12826–12834 (2014).
12. Bonhôte, P., Dias, A.-P., Papageorgiou, N., Kalyanasundaram, K. & Grätzel, M. Hydrophobic, highly conductive ambient-temperature molten salts. *Inorg. Chem.* **35,** 1168–1178 (1996).
13. Visser, A. E., Swatloski, R. P., Reichert, W. M., Griffin, S. T. & Rogers, R. D. Traditional extractants in nontraditional solvents: Groups 1 and 2 extraction by crown ethers in room-temperature ionic liquids. *Ind. Eng. Chem. Res.* **39,** 3596–3604 (2000).
