# Supplementary material for: Minimizing the electrosorption of water from humid ionic liquids on electrodes
Source: Nat Commun. 2018 Dec 4;9:5222. doi: 10.1038/s41467-018-07674-0 (PMC6279789; doi:10.1038/s41467-018-07674-0)
Supplement: Supplementary file 3 — Description of Additional Supplementary Files [file 41467_2018_7674_MOESM3_ESM.pdf]

Description of Additional Supplementary Files

Supplementary Movie 1:

Molecular dynamics simulation of water and room temperature ionic liquid (RTIL) [pyr14][TFSI], starting from water "randomly mixed" with [pyr14][TFSI], mimics the process of demixing, in which water and RTIL are being separated into two phases.

Supplementary Movie 2:

Molecular dynamics simulation of water and RTIL [BMIM][BF_4], starting from water "initially separated" from [BMIM][BF_4], exhibits that water and RTIL spontaneously mix with each other.
